# An electrochemical cell for in operando ^13^C nuclear magnetic resonance investigations of carbon dioxide/carbonate processes in aqueous solution

**DOI:** 10.5194/mr-2-265-2021

**Published:** 2021-05-06

**Authors:** Sven Jovanovic, P. Philipp M. Schleker, Matthias Streun, Steffen Merz, Peter Jakes, Michael Schatz, Rüdiger-A. Eichel, Josef Granwehr

**Affiliations:** 1 Institute of Energy and Climate Research, Fundamental Electrochemistry (IEK-9), Forschungszentrum Jülich, Jülich, Germany; 2 Institute of Technical and Macromolecular Chemistry, RWTH Aachen University, Aachen, Germany; 3 Department of Heterogeneous Reactions, Max Planck Institute for Chemical Energy Conversion, Mülheim an der Ruhr, Germany; 4 Central Institute of Engineering and Analytics, Electronic Systems (ZEA-2), Forschungszentrum Jülich, Jülich, Germany; 5 Institute of Physical Chemistry, RWTH Aachen University, Aachen, Germany

## Abstract

In operando nuclear magnetic resonance (NMR) spectroscopy is one method for the online investigation of electrochemical systems and reactions. It allows for real-time observations of the formation of products and intermediates, and it grants insights into the interactions of substrates and catalysts. An in operando NMR setup for the investigation of the electrolytic reduction of ${\mathrm{CO}}_{2}$ at silver electrodes has been developed. The electrolysis cell consists of a three-electrode setup using a working electrode of pristine silver, a chlorinated silver wire as the reference electrode, and a graphite counter electrode. The setup can be adjusted for the use of different electrode materials and fits inside a 5 mm NMR tube. Additionally, a shielding setup was employed to minimize noise caused by interference of external radio frequency (RF) waves with the conductive components of the setup. The electrochemical performance of the in operando electrolysis setup is compared with a standard ${\mathrm{CO}}_{2}$ electrolysis cell. The small cell geometry impedes the release of gaseous products, and thus it is primarily suited for current densities below 1 mA cm ${}^{-2}$ . The effect of conductive components on ${}^{13}$ C NMR experiments was studied using a ${\mathrm{CO}}_{2}$ -saturated solution of aqueous bicarbonate electrolyte. Despite the $B_{0}$ field distortions caused by the electrodes, a proper shimming could be attained, and line widths of ca. 1 Hz were achieved. This enables investigations in the sub-Hertz range by NMR spectroscopy. High-resolution ${}^{13}$ C NMR and relaxation time measurements proved to be sensitive to changes in the sample. It was found that the dynamics of the bicarbonate electrolyte varies not only due to interactions with the silver electrode, which leads to the formation of an electrical double layer and catalyzes the exchange reaction between ${\mathrm{CO}}_{2}$ and ${\mathrm{HCO}}_{3}^{-}$ , but also due to interactions with the electrochemical setup. This highlights the necessity of a step-by-step experiment design for a mechanistic understanding of processes occurring during electrochemical ${\mathrm{CO}}_{2}$ reduction.

## 1 Introduction

The anthropologically driven atmospheric ${\mathrm{CO}}_{2}$ increase is considered one of the major contributions to global warming [Bibr bib1.bibx20]. A decline in anthropological ${\mathrm{CO}}_{2}$ emissions is seen as improbable due to socio-economic factors [Bibr bib1.bibx18]. Thus, the recycling of ${\mathrm{CO}}_{2}$ by electrochemical conversion to energy-rich materials is of particular interest [Bibr bib1.bibx30]. One promising method in terms of cost and variability is the electrolytic reduction of ${\mathrm{CO}}_{2}$ , usually performed in an aqueous bicarbonate electrolyte solution [Bibr bib1.bibx19]. Depending on the metal electrode, ${\mathrm{CO}}_{2}$ electrolysis yields a number of products, e.g., formate, hydrocarbons, alcohols, and carbon monoxide [Bibr bib1.bibx25]. CO , which is a versatile educt for the chemical industry, e.g., as feedstock for the Fischer–Tropsch process, is obtained by using silver or gold electrodes [Bibr bib1.bibx24].

Despite vivid research, the reaction pathways of electrochemical ${\mathrm{CO}}_{2}$ reduction are still not fully understood [Bibr bib1.bibx25]. There are two main issues, where one originates in the complex equilibrium of ${\mathrm{CO}}_{2}$ and carbonate species in aqueous systems depending on pH, temperature, and partial pressure. These parameters vary with time during the electrolysis and are also a function of distance from the electrode surface [Bibr bib1.bibx25]. The second issue is the electrolytic ${\mathrm{CO}}_{2}$ reduction which suffers further from high overpotentials, mitigated by a few selected metal catalysts. There is evidence that the formation of an intermediate ${\mathrm{CO}}_{2}^{-}$ radical can cause an overpotential [Bibr bib1.bibx25].

To gain insights into the dynamic processes of an electrochemical system, it is imperative to monitor the complete system during operation, e.g., using in operando spectroscopic techniques [Bibr bib1.bibx4]. Nuclear magnetic resonance (NMR) spectroscopy is a flexible and powerful method for reaction monitoring or quantitative chemical analysis [Bibr bib1.bibx53]. The NMR study of batteries is often associated with broad line widths inherent to solid-state materials. For in operando investigations of liquid-state electrolysis systems, high spectral resolution is a critical factor. The determination of structural information for small molecules relies on the detection of minor changes in chemical shifts and J couplings in the range of a few Hertz. Thus, signal line widths have been of major concern since the earliest publications in this area of research. Several experiment setups for the electrochemical reduction and/or oxidation of organic molecules are suggested in the literature [Bibr bib1.bibx12]. The first in operando flow cell for the investigation of electrochemical processes consisted of a two-electrode setup inside a 5 mm NMR tube [Bibr bib1.bibx46]. A Pt/Hg wire working electrode outside the sensitive volume was placed inside a 3 mm tube concentric to the NMR tube. At the bottom of the 3 mm tube, a capillary released the reaction products into the sensitive volume of the 5 mm tube. The setup allowed sample spinning, which was required due to the low spectral resolution of the spectrometer at that time. This approach was then adapted for the investigation of anion radical decays and improved by using three electrodes [Bibr bib1.bibx37].

An alternative in operando setup employed thin-film electrodes to minimize distortions of the $B_{0}$ and $B_{1}$ field by conductive parts of the electrolysis cell [Bibr bib1.bibx41], where ${}^{1}$ H line widths of 0.9 Hz could be achieved [Bibr bib1.bibx45]. Additionally, radio frequency (RF) chokes were introduced to this setup to minimize interactions between NMR and the potentiostat [Bibr bib1.bibx55]. However, manufacturing of thin-film electrodes is not easily adaptable. An alternative setup was constructed with improved accessibility [Bibr bib1.bibx32]. The electrolysis cell employed carbon fiber electrodes with a high surface area and could be set up without the need for special equipment [Bibr bib1.bibx13]. However, the use of carbon fiber electrodes limits the variety of possible electrocatalysts.

A different technique for the coupling of electrochemistry and magnetic resonance is hyphenated electrochemical NMR [Bibr bib1.bibx2], where the electrochemical cell is physically separated from the NMR spectrometer by passing the electrochemically generated species to an NMR probe by flow. This technique does not suffer from $B_{0}$ and $B_{1}$ distortions, but there is a time delay between generation and detection of the electrochemical species due to the physical separation.

To directly monitor paramagnetic species, electron paramagnetic resonance (EPR) spectroscopy was employed in a recent study as a screening tool for electrocatalysts [Bibr bib1.bibx43].

Despite their first appearance in 1975, electrochemical in operando NMR investigations of liquid-state systems are tested primarily on well-studied, simple redox systems. More recently in operando NMR has been used to study biological systems [Bibr bib1.bibx58]. However, this method has not yet been utilized for the investigation of industrial and energy applications, e.g., the electrolytic reduction of ${\mathrm{CO}}_{2}$ .

The majority of research was performed using ${}^{1}$ H NMR due to the high sensitivity compared to other nuclei, with only few attempts made to investigate ${}^{13}$ C systems [Bibr bib1.bibx2]. ${}^{13}$ C NMR offers a high spectral width and thus allows for an increased separation between signals, but suffers from a low natural abundance of the nucleus. To increase the sensitivity, steady-state free precession (SSFP) was suggested to achieve a high signal-to-noise ratio (SNR) for short measurement times despite using non-enriched samples [Bibr bib1.bibx44].

In operando ${}^{13}$ C NMR spectroscopy is ideally suited for studying the electrolytic reduction of ${\mathrm{CO}}_{2}$ to CO , which requires high resolution to monitor changes in the structure of the educt and the ability to use high-sensitivity NMR equipment. To investigate processes of interest directly, the working electrode needs to be placed in the sensitive volume of the NMR coil. On the other hand, conductive components in the sample can lead to distortions of $B_{0}$ and $B_{1}$ . These effects can be minimized by proper placement and orientation of the electrode and by pulse sequences that are robust against $B_{0}$ and $B_{1}$ field distortions [Bibr bib1.bibx21]. For a versatile cell setup, ease of construction, adaptability for various metal electrodes, and the applicability in unmodified NMR liquid-state probes is desirable.

This work presents an electrolysis cell for the in operando NMR investigation of electrolytic ${\mathrm{CO}}_{2}$ reduction. The cell is constructed inside a 5 mm NMR tube and consists of a three-electrode setup, which can easily be adapted. The electrochemical performance of the setup was evaluated by characterizing ${\mathrm{CO}}_{2}$ in a 1 M ${\mathrm{KHCO}}_{3}$ electrolyte with a) all necessary electrochemical equipment connected and b) without connection. To investigate the mobility and interactions of the reactant and the electrolyte, $T_{1}$ , $T_{2}$ and exchange time constants between ${\mathrm{CO}}_{2}$ and ${\mathrm{HCO}}_{3}^{-}$ were determined.

## 2 In operando NMR setup

### 2.1 Electrolysis cell

The three-electrode electrolysis cell fits a standard 5 mm NMR tube and consists of a 2.5 mm × 4 mm × 0.05 mm silver foil (GoodFellow, Hamburg, Germany) with an area of 10 mm ${}^{2}$ as a working electrode and a graphite rod with 1 mm diameter and 50 mm length (GoodFellow, Hamburg, Germany) as a counter electrode. A chlorinated silver wire tip with a diameter of 0.25 mm (GoodFellow, Hamburg, Germany) was employed as a micro Ag / AgCl reference electrode. All electrodes were connected using silver wire with a diameter 0.25 mm, insulated with polytetrafluoroethylene (PTFE) of 0.024 mm thickness (GoodFellow, Hamburg, Germany). A graphite counter electrode prevents the dissolution of metals during electrolysis and which may deposit at the working electrode, resulting in a change in catalytic properties [Bibr bib1.bibx7]. This process becomes pronounced for small setups with half-cell reactions not separated by a membrane since species originating at the counter electrode diffuse sufficiently quickly towards the working electrode.

To join the silver lead wire and the silver foil, the wire insulation was stripped off over a length of about 1–2 mm. The skinned wire tip was pressed onto the silver foil while heating to 450 ${}^{\circ }$ C for a few seconds using a soldering iron. The counter electrode was connected by soldering where ca. 2 cm of the silver wire insulation was removed and wrapped around one end of the graphite rod.

The reference electrode was prepared by cleaning the stripped tip (ca. 2 mm) of a silver wire in concentrated nitric acid for 30 s. The electrode was subsequently transferred into a 1 M aqueous solution of potassium chloride ( ≥ 99.5 % purity; Sigma Aldrich, Munich, Germany) for 30 min. During this process a thin layer of silver chloride (AgCl) formed, creating a micro Ag / AgCl reference electrode [Bibr bib1.bibx29]. The averaged potential of the micro Ag / AgCl electrode was determined to be 0.132±0.004 V vs. a commercial Ag / AgCl (3 M KCl) reference electrode in 1 M ${\mathrm{KHCO}}_{3}(\mathrm{aq})$ . Potentials provided in this work are given vs. the micro Ag / AgCl electrode. The commercial electrode was specified with a potential of 0.210 V vs. a normal hydrogen electrode (NHE). Thus, the potential of the micro Ag / AgCl reference electrode was determined as of 0.342±0.004 V vs. NHE in ${\mathrm{CO}}_{2}$ -saturated 1 M ${\mathrm{KHCO}}_{3}$ . The potential of the micro reference electrode was constant during one experiment but may vary slightly in different chemical environments.

**Figure 1 Ch1.F1:**
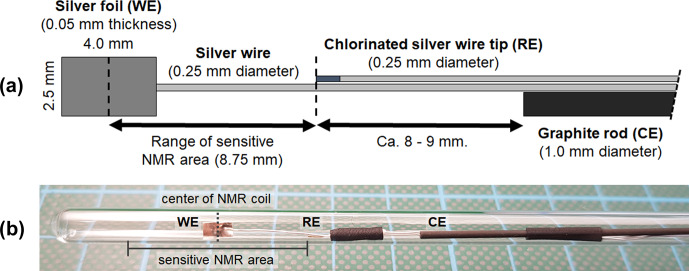
**(a)** Geometry and arrangement of the three-electrode in operando NMR setup. It consists of a silver foil working electrode (WE), a graphite rod counter electrode (CE), and a micro Ag / AgCl reference electrode (RE). The reference electrode was placed on the edge of the sensitive NMR area to minimize the amount of conductive material during NMR measurements while maintaining a small ohmic potential drop between the working and reference electrodes. **(b)** Photograph of the electrode setup inside a 5 mm tube.

The electrodes were arranged in a geometry as shown in Fig. [Fig Ch1.F1]a and fixed using PTFE tape and a heat shrink tubing. The distance between the center of the working electrode and the reference electrode was adapted to the height of the sensitive volume of the NMR coil. The position of the working electrode inside the 5 mm tube was adjusted to match the center of the coil. This arrangement minimizes the content of conductive material inside the NMR coil, thus reducing distortions of $B_{0}$ and interactions with $B_{1}$ . Additionally, a minimized distance between the reference and working electrodes ensures a small uncompensated resistance of 5±2 Ω and correspondingly a small internal resistance (iR) drop for all electrochemical measurements. An iR drop is a drop in the potential for an electrochemical system caused by the uncompensated resistance according to Ohm's law. Thus, the iR drop is proportional to the uncompensated resistance and the applied current.

The lead wires of the electrodes were passed through a drilled opening of an NMR tube cap. Cellulose nitrate glue (UHU HART, UHU, Bühl, Germany) was applied to the top of the tube cap and the protruding connection wires for mechanical stability. The glue fixes the position of the electrodes inside the 5 mm tube and seals the drilled opening in the cap. Additionally, ethyl cyanacrylate glue (Loctite 406, Henkel, Düsseldorf, Germany) was applied on the top after the cellulose nitrate glue hardened in order to decrease the gas permeability.

### 2.2 Cell holder

The holder for the electrolysis cell is shown in Fig. [Fig Ch1.F2]. The setup enables an easy and stable connection between the thin silver wires of the cell and the shielded coaxial cables of the potentiostat. Furthermore, it increases the structural stability of the cell by reducing the weight and strain as well as vibrations of the coaxial cables. The frame of the cell holder was 3D-printed using acrylnitrile butadiene styrene (ABS) copolymer (Filamentworld, Neu-Ulm, Germany). For each electrode a non-magnetic SMA coaxial connector (model 23_SMA-50-0-13/111_NE, Huber+Suhner, Herisau, Switzerland) was fixed to the frame using non-magnetic screws. To connect the electrolysis cell, the silver wires were soldered to the connector pins. The bottom hole of the cell holder was adjusted to the outer diameter of the NMR tube plus the tube cap. The 5 mm tube containing the electrolysis cell is mounted into the cell holder from the top opening, and the cell is fixed by tightly squeezing the tube cap at the top end of the NMR tube into the bottom hole of the holder.

**Figure 2 Ch1.F2:**
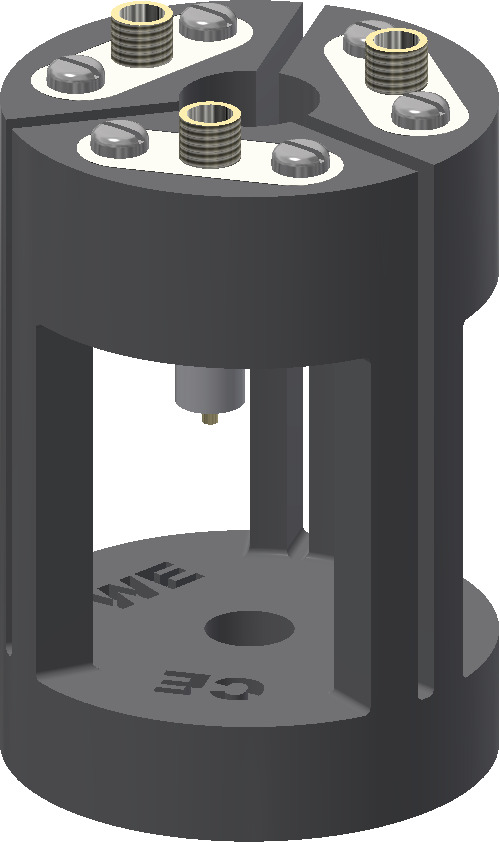
Cell holder consisting of the 3D-printed frame (black) and three SMA coaxial connectors (white and gold). The electrolysis cell is fixed inside the cell holder and the electrode wires are soldered to the pins of the SMA coaxial connectors.

The direct insertion of the in operando cell into the probe was found to be mechanically unstable. To stabilize the sample inside the magnet and to achieve a mechanical separation of probe and cell, a dismounted turbine of a magnet lift was fixed on top of the probe. A spinner (Bruker, Germany) matching the opening of the turbine was then attached to the 5 mm tube of the in operando cell. The sample with the attached spinner was inserted into the turbine and probe by hand. The vertical position of the in operando cell inside the spinner was adjusted to match the sensitive NMR volume. No sample spinning was performed.

### 2.3 Assembly for noise depression

The in operando cell was connected to a potentiostat using shielded coaxial cables with SMA connectors. The top opening of the magnet was closed with a copper plate containing two RF feedthroughs for potentiostat connection (NMR Service, Erfurt, Germany). Additionally, three low-pass RF filters (SLP-5+, SLP-15+, SLP-30+, Mini Circuits, New York, USA) were connected to each cable for noise depression (Fig. [Fig Ch1.F3]). The SLP-5+ low-pass filter ( <5 MHz) was connected to the copper plate connection at the top of the magnet, and the SLP-15+ ( <15 MHz) & SLP-30+ ( <30 MHz) filters were attached to the potentiostat connections. Since the connections for the potentiostat are unshielded, a silver cloth was wrapped around all unshielded cables. In addition, the body of the probe, the NMR magnet, and the potentiostat were connected to a common ground. The shielding setup is shown in Fig. [Fig Ch1.F3].

**Figure 3 Ch1.F3:**
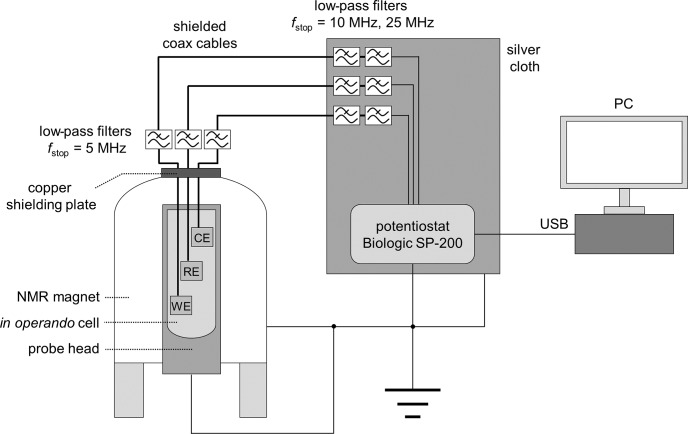
Schematic drawing of the in operando NMR electrolysis setup with shielding, RF filters, and potentiostat.

### 2.4 Simulation of the $B_{1}$ field and the nutation behavior within the in operando cell

The distortion of the $B_{1}$ field in the proximity of the metal electrode was numerically simulated using EMpro (Version 2020, Keysight Technologies). A square Helmholtz coil consisting of two parallel square-shaped wires with a distance of 0.5445 times the length for each side of the square was designed to mimic a homogeneous RF field in the vicinity of the electrode. An ideal conductor served as coil material, and both squares of the coil were driven synchronously by a current source. The silver electrode was placed in the center of the coil as shown in Fig. [Fig Ch1.F4]d. The simulation was performed for three different angles (0, 45, 90 ${}^{\circ }$ ) between the $B_{1}$ field and the electrode plane and data points were acquired with a resolution of 0.4 mm. The complex magnetic field vectors of the simulated volume were exported by means of a Python script (Python 3.7, Python Software Foundation) for data processing.

**Figure 4 Ch1.F4:**
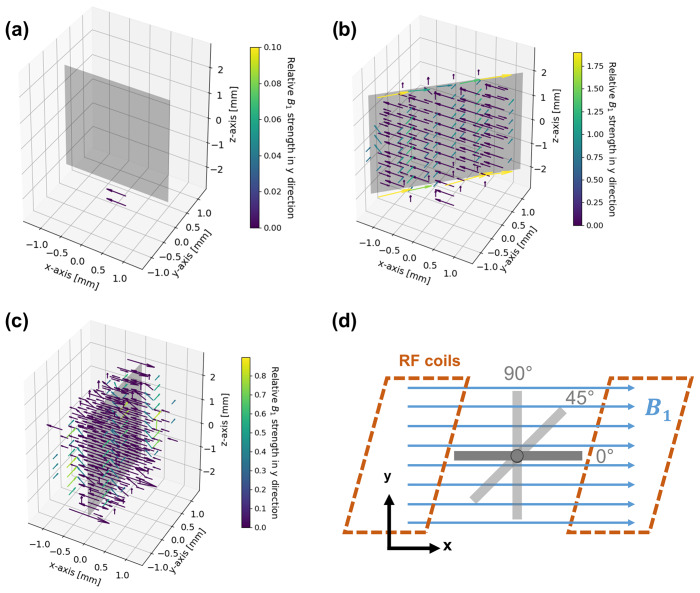
$B_{1}$ field simulation in the proximity of the metal electrode for angles of 0 ${}^{\circ }$ **(a)**, 45 ${}^{\circ }$ **(b)**, and 90 ${}^{\circ }$ **(c)** between the direction of the incoming RF field and the electrode surface, and geometry and arrangement of the metal electrode in relation to the $B_{1}$ field in the simulations **(d)**. The incoming RF field points towards the positive x axis. The vectors represent the deviations in field strength and direction compared to the undistorted RF field. Deviations smaller than $B_{1}/e$ are not shown in order to increase clarity. For a better visibility of phase deviations, the vectors are color coded according to their relative field strength in the y direction compared to the incoming field amplitude. Note that all figures have individual color-bar ranges. No distortion is present for a parallel (0 ${}^{\circ }$ ) orientation of the RF field and electrode. The angled (45 ${}^{\circ }$ ) and perpendicular (90 ${}^{\circ }$ ) orientations cause major distortions in immediate proximity to the electrode, which diminish at a distance of 0.6–0.8 mm.

Eddy currents caused by the oscillating $B_{1}$ field are formed at the metal surface (Fig. [Fig Ch1.F4]). In turn, the eddy currents induce a magnetic field that distorts amplitude and phase of the excitation pulse. The distortion of the $B_{1}$ field strongly depends on the angle between the electrode and the RF field [Bibr bib1.bibx9]. For a parallel configuration, i.e. at an angle of 0 ${}^{\circ }$ , distortions of the $B_{1}$ field are minimized (Fig. [Fig Ch1.F4]a). Correspondingly, there is only a small eddy current formation due to the minimal surface area remaining perpendicular to the $B_{1}$ field.

For a perpendicular (90 ${}^{\circ }$ ) orientation of the electrode (Fig. [Fig Ch1.F4]c) the $B_{1}$ field showed major distortions, which lead to a decrease in amplitude of the $B_{1}$ field in the proximity of the electrode surface. However, the $B_{1}$ field strength is increased at the top and bottom edges of the electrode. At the side edges, the direction of the field, i.e. the phase of $B_{1}$ , changed. At a distance of about 0.8 mm from the electrode surface, the strength of the RF distortions decreased to 1/e of the $B_{1}$ field.

Smaller distortions of $B_{1}$ were observed for the 45 ${}^{\circ }$ orientation of the electrode (Fig. [Fig Ch1.F4]b), affecting mostly the direction of the field, whereas the signal amplitude decreased at the surface. Major distortions of the $B_{1}$ phase were present along the top and bottom edges of the electrode. The distortions decreased to 1/e of the $B_{1}$ field strength at a distance of ca. 0.6 mm from the electrode surface.

It can therefore be concluded that an electrode orientation planar to $B_{1}$ is considered optimal. Amplitude and phase of the $B_{1}$ field distortions depend on the spatial orientation. However, distortions are significant in the immediate proximity of the electrode (0.6–0.8 mm from the surface). Therefore, the majority of the volume inside a 5 mm NMR tube can be regarded as free of distortions for the chosen electrode geometry and thus can be probed by NMR spectroscopy without additional measures. For the current setup a minute adjustment of the orientation is not necessary. It should be noted that the distortions of the $B_{1}$ field depend on orientation and on electrode size, and adjustments can be required for larger in operando electrolysis cells.

**Figure 5 Ch1.F5:**
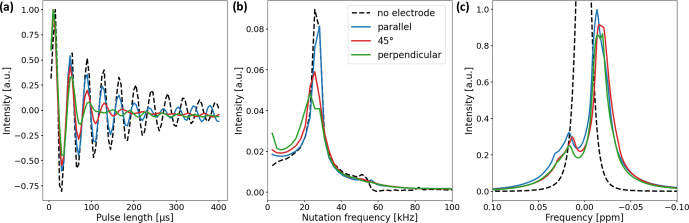
**(a)** Nutation curves of the ${}^{1}$ H water resonance using the in operando cell with electrode orientations of 0 ${}^{\circ }$ (blue), 45 ${}^{\circ }$ (red), and 90 ${}^{\circ }$ (green). The nutation curve of a water sample without an electrode is shown for comparison (black dashed line). Deviations from the undistorted nutation curve are largest for the perpendicular electrode orientation and minimal for the parallel orientation. **(b)** Fourier transform of the nutation curves. The main component of the undistorted sample nutates at a frequency of 25.6 kHz (15 µ s 90 ${}^{\circ }$ pulse length). For the samples with electrode setups, the width of the main component increases and a low-frequency component appears. **(c)** ${}^{1}$ H water NMR spectrum with and without electrode setup. The $B_{0}$ field was not shimmed after electrode insertion. The signal shape is mainly governed by $B_{0}$ field distortions and only slightly affected by deviations in the $B_{1}$ field.

Two experiments were performed to validate the $B_{1}$ field simulations and study the effect of $B_{1}$ field distortions on NMR measurements. First, a nutation experiment using distilled water was performed using a Bruker Avance III HD spectrometer with a 9.4 T wide-bore magnet (400 MHz ${}^{1}$ H RF frequency) and a broadband gradient probe (Bruker DiffBB). The experiment was conducted with and without the electrode setup where the electrode orientation was either parallel, perpendicular, or at a 45 ${}^{\circ }$ angle with respect to $B_{1}$ . Data points were acquired at a constant pulse power of 13.9 W for pulse lengths between 5 and 400 µ s using 5 µ s steps. Nutation curves were acquired using the ${}^{1}$ H water peak due to the higher S/N ratio compared to the ${}^{13}$ C resonances of ${\mathrm{HCO}}_{3}^{-}$ and ${\mathrm{CO}}_{2}$ (Fig. [Fig Ch1.F5]a). Figure [Fig Ch1.F5]b shows the Fourier transform of the nutation curves. Secondly, the effect of the $B_{1}$ distortions on the water signal shape was studied. In Fig. [Fig Ch1.F5]c the NMR signal shapes of water for different electrode orientations are compared. The $B_{0}$ field was shimmed on a sample containing pure distilled water without the electrode setup and not reshimmed after insertion of the electrodes in order to examine the distortion of the $B_{0}$ field by the metal components.

As predicted by the simulations, deviations in the nutation curves are largest for the perpendicular electrode orientation and minimal for the parallel orientation compared to the neat nutation behavior. The magnitude of deviations for the 45 ${}^{\circ }$ electrode orientation is in between the parallel and perpendicular orientations. The nutation curve for the perpendicular orientation exhibits both the fastest decay and the broadest distribution of nutation frequencies. This becomes evident in Fig. [Fig Ch1.F5]b, where the perpendicular orientations feature a broad main component distributed around 25.6 kHz, with an additional component at low frequencies.

The 90 and 180 ${}^{\circ }$ pulse lengths of all three electrode orientations exhibit minor deviations compared to the pulse lengths of the pure water sample. Therefore, common pulse sequences can be applied for studies using the in operando cell. This is evident in Fig. [Fig Ch1.F5]c, where only small differences due to $B_{0}$ field distortions between the unshimmed water signals for different electrode orientations are apparent.

All simulations and NMR measurements on the orientation-dependent magnetic field distortions caused by metallic components are in line with the literature [Bibr bib1.bibx28]. In these studies an electrode orientation parallel to the $B_{1}$ field direction is considered optimal. Strong $B_{1}$ field distortions as well as signal loss in the proximity of the metal are observed for perpendicular orientations. It was also shown that angled orientations lead to amplification of the $B_{1}$ field along the metal boundaries, which is consistent with the pronounced distortions of $B_{1}$ field strength and phase alterations observed for the 45 ${}^{\circ }$ electrode orientation.

## 3 Materials and methods

A 1 M aqueous solution of 98 % ${}^{13}$ C-enriched ${\mathrm{KHCO}}_{3}$ (Sigma Aldrich, Munich, Germany) was used as an electrolyte. The electrolyte was pre-chilled inside a polyethylene vial in a 10 ${}^{\circ }$ C water bath. Ca. 1 mL of chilled electrolyte was filled into a 5 mm NMR tube and saturated with 99 % ${}^{13}$ C-enriched ${\mathrm{CO}}_{2}$ (Cambridge Isotope Laboratories, Tewksbury, USA) by bubbling for 20 min at a temperature of 10 ${}^{\circ }$ C if not stated otherwise. The ${\mathrm{CO}}_{2}$ was bubbled into the electrolyte using a 1/16 inch PEEK tube, and the flow rate was adjusted to ca. 0.3 mL s ${}^{-1}$ . The three-electrode setup was placed inside the 5 mm tube filled with ${\mathrm{CO}}_{2}$ -saturated electrolyte, ensuring that the contact between counter electrode and silver wire was not immersed in liquid. Prior to sealing, the gas phase inside the tube was aerated with ${}^{13}$ C-labeled ${\mathrm{CO}}_{2}$ gas. All preparation steps were performed under ambient conditions.

The electrochemical experiments were performed using a BioLogic SP-200 potentiostat (BioLogic Science Instruments, Seyssinet-Pariset, France) at a temperature of 10 ${}^{\circ }$ C, controlled by a surrounding water bath. The electrochemical performance of the in operando cell was evaluated using chronopotentiometry (CP) at several current densities up to 4 mA cm ${}^{-2}$ for 15 min each and linear sweep voltammetry (LSV) in the range of - 1.0 to - 2.5 V vs. Ag / AgCl (rate 10 mV s ${}^{-1}$ ) afterwards. Between the electrochemical experiments the system was allowed to relax for 5 min. An equivalent chronopotentiometry experiment was performed using a 1 cm ${}^{2}$ silver electrode and reference and counter electrodes of identical size and material. This experiment serves as a reference. The reference chronopotentiometry experiment was performed in a cleaned glass beaker filled with 60 mL of aqueous ${\mathrm{CO}}_{2}$ -saturated 1 M ${\mathrm{KHCO}}_{3}$ electrolyte, denoted as bulk cell. Working and counter electrodes were arranged in a parallel geometry inside the bulk cell. All distances between working and counter electrodes and between the working and reference electrodes are identical to the in operando cell.

The potential of the micro reference was determined vs. a commercial Ag / AgCl reference electrode with a double-junction system and a 3 M aqueous KCl bridge electrolyte. The measurement was performed using the electrolyte of the ${\mathrm{CO}}_{2}$ electrolysis where the reference electrode potential was averaged over 10 min. Both reference electrodes were equilibrated for 10 min prior to the experiment.

All ${}^{13}$ C NMR measurements were performed using a Bruker Avance III HD spectrometer with a 14.1 T wide-bore magnet (150.9 MHz RF frequency for ${}^{13}$ C) and a broadband gradient probe (Bruker DiffBB); 90 ${}^{\circ }$ pulses were achieved using a pulse length of 15.5 µ s and a pulse power of 58.7 W, and the relaxation delay was set to 85 s. Spectra were post-processed by applying a 1 Hz line broadening. NMR experiments were performed at a temperature of 10 ${}^{\circ }$ C if not stated otherwise. Concentrations of the carbon species in the ${\mathrm{CO}}_{2}$ -saturated electrolyte with ${\mathrm{CO}}_{2}$ atmosphere were determined in a sealed NMR tube using sodium trimethylsilylpropanesulfonate (DSS) (Sigma Aldrich, Munich, Germany) as a reference ( c (DSS) = 61.62 mM) and a ${}^{1}$ H WALTZ-16 sequence for decoupling [Bibr bib1.bibx51]. The chemical shift scale of all ${}^{13}$ C spectra was referenced to the frequency offset of DSS. DSS was not employed for in operando experiments because the organic salt can alter the electrochemical behavior. The ${\mathrm{CO}}_{2}$ -saturated electrolyte was examined using longitudinal, $T_{1}$ , and transverse, $T_{2}$ , relaxation and exchange time measurements. $T_{1}$ relaxation time constants were determined using a saturation recovery pulse sequence with equispaced saturation pulses using logarithmically spaced recovery times between 1 and 128 s. Transverse relaxation time constants were determined using a Carr–Purcell–Meiboom–Gill (CPMG) pulse sequence with an echo time of 5 ms [Bibr bib1.bibx14]. The exchange time between ${\mathrm{HCO}}_{3}^{-}$ and solvated ${\mathrm{CO}}_{2}$ was assessed by a 1D exchange spectroscopy (EXSY) sequence [Bibr bib1.bibx3], which uses a shaped Gauss pulse with 100 Hz bandwidth for the selective inversion of the bicarbonate resonance at 160.7 ppm. The center frequency of the selective inversion pulse was adjusted in case of a ${\mathrm{HCO}}_{3}^{-}$ frequency shift.

The exchange time constant $T_{\text{exc}}$ was determined by fitting the evolution of the ${\mathrm{CO}}_{2}$ signal integral I ( ${\mathrm{CO}}_{2}$ ) as a function of the mixing time $\tau_{\text{m}}$ to

1 $$\begin{array}{rl} I({\mathrm{CO}}_{2}) & =I_{0}({\mathrm{CO}}_{2})\left\{1-2\left[\mathrm{exp}\left(-\frac{\tau_{\text{m}}}{T_{\text{exc}}+T_{1}}\right)\right.\right.\\ & \left.\left.-\mathrm{exp}\left(-\frac{\tau_{\text{m}}}{T_{1}}\right)\right]\right\}, \end{array}$$

where $I_{0}$ is the signal integral at $\tau_{\text{m}}=0$ . This simplified fitting equation is valid under the conditions that the bicarbonate concentration substantially exceeds the ${\mathrm{CO}}_{2}$ concentration and both species possess identical $T_{1}$ times [Bibr bib1.bibx3].

## 4 Results and discussion

### 4.1 Electrochemical performance of the in operando electrolysis cell

The time-dependent potential curves for the chronopotentiometry measurements are shown in Fig. [Fig Ch1.F6]a. The potentials observed for both the in operando and the bulk cell are within the range reported in the literature, as the values depend on the properties of the catalyst and the electrolysis cell. ${\mathrm{CO}}_{2}$ electrolysis starts at - 1.33 V vs. Ag / AgCl for the in operando cell and at - 1.23 V vs. Ag / AgCl for the bulk cell. However, three types of deviations could be deduced from the potential curves for the in operando cell compared to the bulk cell. First, higher overpotentials are observed. Second, it took longer for the in operando cell to equilibrate when the current is applied and switched off. Third, increasing oscillations in the potential and additional noise are observed, starting at - 1 mA cm ${}^{-2}$ .

**Figure 6 Ch1.F6:**
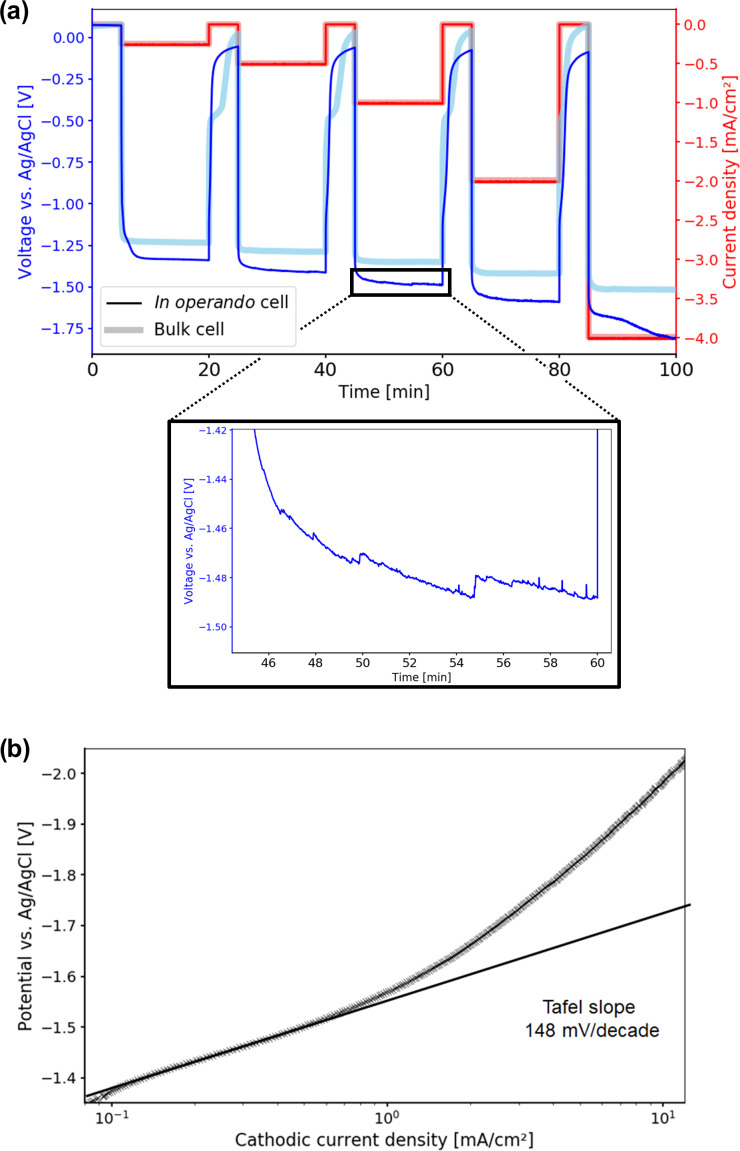
**(a)** Time-dependent potential curves during the chronopotentiometry measurement. Electrolytic reduction of ${\mathrm{CO}}_{2}$ starts at - 1.33 V vs. Ag / AgCl for the in operando cell. Compared to the bulk cell, higher overpotentials are observed. Starting at 1 mA cm ${}^{-2}$ , oscillations and increased noise appear, which are caused by stuck product gas bubbles. **(b)** Tafel plot of the electrolytic ${\mathrm{CO}}_{2}$ reduction in the in operando electrolysis cell. The Tafel slope was determined in the low current density region as 148 mV per decade, resulting in a transfer coefficient of 0.38 at 10 ${}^{\circ }$ C.

The non-parallel geometry of the electrodes in the in operando cell may be causing the first two deviations. For a parallel geometry the distance between the working electrode and the counter electrode is approximately constant across the whole WE surface. Thus, the iR drop is constant across the electrode, and the surface potential is uniform. However, in a non-parallel geometry, there is a spatially dependent iR drop between the working and counter electrodes, which leads to non-uniform potential distribution across the electrode surface. As a result, electrolysis preferably takes place in the closest region between the working and counter electrodes, where the iR drop is minimal. The ${\mathrm{CO}}_{2}$ concentration within that region decreases during electrolysis, and thus the concentration overpotential increases. When the concentration overpotential exceeds the increase in iR drop for a more distant region, the electrolytic process shifts to that location.

For the electrode setup used in the in operando cell, the edge of the silver sheet is the region of the working electrode closest to the counter electrode. For a current density of - 0.25 mA cm ${}^{-2}$ , the electrolytic ${\mathrm{CO}}_{2}$ reduction at the silver foil edge takes place at an identical overpotential compared to the bulk cell. However, because of the small area the electrolysis at the electrode edge is not sustainable, and therefore the diffusive ${\mathrm{CO}}_{2}$ transport becomes limited. As a result, the ${\mathrm{CO}}_{2}$ concentration is depleted after 2 min of electrolysis. At this point, ${\mathrm{CO}}_{2}$ reduction takes place at the next-nearest region of the counter electrode, a portion of the silver foil plane, where ${\mathrm{CO}}_{2}$ is more readily regenerated by diffusion. However, the iR drop at the silver foil plane is increased compared to the edge, and thus the potential decreases.

With increasing current density, ${\mathrm{CO}}_{2}$ conversion increases. Therefore an increasing area of the silver electrode surface with an increasing distance to the counter is used for reduction. This results in a rising iR drop and a further increasing overpotential compared to the bulk cell. This effect may also cause the instability in potential of the in operando cell at a current density of - 4 mA cm ${}^{-2}$ . It is important to separate this effect from the expected increase in concentration overpotential with increasing current density, which was also observed for the bulk cell.

The oscillations and increased noise observed for the potential curve of the in operando cell at higher current densities are caused by the formation of gaseous products, i.e., CO and $H_{2}$ , in the confined cell geometry. The gas bubbles tend to stick to the glass walls, the electrodes or the connection wires, until they reach a sufficient size to detach and rise to the top. Diameters up to one-third of the size of the electrode surface were observed for the gas bubbles. These bubbles blocked significant fractions of the electrodes, thus affecting the electrochemical measurements. For the bulk cell, only a small percentage of gas bubbles adsorbed on the electrodes or the cell due to the larger distance between the electrodes and the glass walls. Moreover, the larger electrode was affected by the comparatively smaller gas bubbles.

For the in operando cell, the Tafel slope was determined as 148 mV per decade from current–voltage (IV) curves of the LSV experiments in the low-current density region as shown in Fig. [Fig Ch1.F6]b. In this region no mass transport limitations for the electrolytic reduction of ${\mathrm{CO}}_{2}$ occurred. The slope of the Tafel plot translates to a charge transfer coefficient of α=0.38 . From the literature, values for the Tafel slope can range from 130 to 140 mV per decade, resulting in charge transfer coefficients of 0.41–0.45 for comparable systems at room temperature [Bibr bib1.bibx26]. The minor discrepancy between measured and literature values may originate from the lower temperature where experiments were performed, which results in a lower thermal energy for the activation of processes and thus lower diffusion rates.

Overall, the in operando cell shows a comparable performance to a bulk electrolysis cell in the low-current density range, i.e., below - 1 mA cm ${}^{-2}$ . Due to the non-parallel cell geometry there is a spatially dependent iR drop distribution across the working electrode surface, which is unfavorable for electrolysis experiments. Since this effect is more pronounced for larger electrode geometries, it is crucial to reduce the working electrode size. However, a larger electrode area is beneficial for minimizing the current density during electrolysis and thus the concentration overpotential. The 10 mm ${}^{2}$ electrode used in the in operando cell represents a compromise between both considerations. Nonetheless, the potential becomes unstable at higher current densities, and electrochemical measurements may be distorted by gas bubbles stuck in the confined glass tube.

### 4.2 NMR evaluation of the in operando electrolysis setup

The ${}^{13}$ C spectrum of the ${\mathrm{CO}}_{2}$ -saturated electrolyte is shown in Fig. [Fig Ch1.F7]a.

**Figure 7 Ch1.F7:**
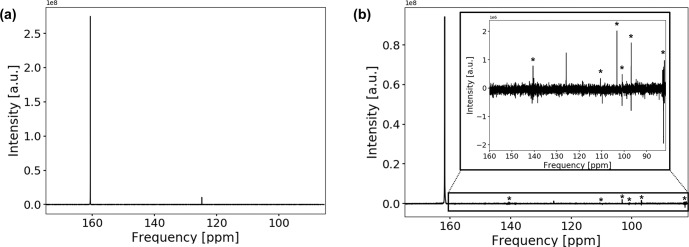
${}^{13}$ C spectrum of the ${\mathrm{CO}}_{2}$ -saturated electrolyte without **(a)** and with **(b)** electrodes. Measurements with electrodes include connection cables and a powered potentiostat but no shielding. The peak positions of bicarbonate and solvated carbon dioxide are at 160.7 and 124.7 ppm, respectively. Peak positions are shifted downfield by about 1.1 ppm when the conductive components are introduced. The spectrum in **(b)** suffers from increased noise as well as from external RF signals, which are comparable in intensity to the ${\mathrm{CO}}_{2}$ signal. External RF signals have been marked with (*).

Both signals in the spectra are assigned to ${\mathrm{HCO}}_{3}^{-}$ at 160.7 ppm and solvated ${\mathrm{CO}}_{2}$ at 124.7 ppm [Bibr bib1.bibx1]. The low solubility of carbon dioxide in water causes a weaker signal compared to bicarbonate. No signal of gaseous carbon dioxide could be observed since the gas phase inside the NMR tube is outside of the sensitive volume of the RF coil. For a measured pH of 8.2±0.15 after ${\mathrm{CO}}_{2}$ aeration of the electrolyte, about 1 % of dissolved carbonate is expected. However, due to the fast exchange of ${\mathrm{HCO}}_{3}^{-}$ and ${\mathrm{CO}}_{3}^{2-}$ , signals for both species coalesced into one peak.

The concentrations of the carbon species in the ${\mathrm{CO}}_{2}$ -saturated electrolyte are shown in Table [Table Ch1.T1]. During aeration of the electrolyte with ${\mathrm{CO}}_{2}$ the concentration of bicarbonate ( ${\mathrm{HCO}}_{3}^{-}$ ) increased by a factor of 2. The measured concentration of ${\mathrm{CO}}_{2}$ is in the uncertainty limits of the theoretical equilibrium concentration for aqueous solution ( $c_{1013\;\mathrm{hPa},\;10\;{}^{\circ }C}=52.7$ mM) [Bibr bib1.bibx48]. The uncertainty is caused by a combination of a weak signal-to-noise ratio of the DSS methyl group reference signal and imperfections of the temperature control ( ≤1 ${}^{\circ }$ C).

**Table 1 Ch1.T1:** Concentrations of carbon species in ${\mathrm{CO}}_{2}$ -saturated electrolyte at 10 ${}^{\circ }$ C and 1013 hPa ${\mathrm{CO}}_{2}$ pressure.

Carbon species	Concentration (M)
${\mathrm{HCO}}_{3}^{-}$	1.82±0.14
${\mathrm{CO}}_{2}$	$(55.0\pm 4.4)\times 10^{-3}$
Total carbon	1.87±0.14

**Table 2 Ch1.T2:** Signal-to-noise ratio of the ${}^{13}$ C ${\mathrm{HCO}}_{3}^{-}$ signal under different conditions.

Temperature	Conditions	Signal-to-noise
( ${}^{\circ }$ C)		ratio (–)
22	No conductive material	1247
	In operando cell	397
	In operando cell and connection cables	290
10	No conductive material	1994
	In operando cell and connection cables	399
	Full in operando electrolysis setup with shielding	2510

As the solubility of ${\mathrm{CO}}_{2}$ in water is low, the ${}^{13}$ C signal of ${\mathrm{CO}}_{2}$ is weak even for using ${}^{13}$ C-labeled and fully saturated samples. It is therefore essential to optimize the signal-to-noise ratio before experiments. To investigate the effect of the in operando setup on the ${}^{13}$ C spectra, the signal-to-noise ratio of the ${\mathrm{HCO}}_{3}^{-}$ signal was determined under different conditions (Table [Table Ch1.T2]). Decreasing the temperature from 22 to 10 ${}^{\circ }$ C significantly improved the signal-to-noise ratio. As ${\mathrm{CO}}_{2}$ shows a higher solubility at lower temperatures (cf. $c_{1013\;\mathrm{hPa},\;22\;{}^{\circ }C}=38.0$ mM) [Bibr bib1.bibx48], the signal-to-noise ratio of the ${\mathrm{CO}}_{2}$ signal increased by a factor of ca. 1.4. The equilibrium constant for the ${\mathrm{CO}}_{2}/{\mathrm{HCO}}_{3}^{-}$ equilibrium changed by only 1 % due to the decrease in temperature. Therefore an increase in ${\mathrm{CO}}_{2}$ concentration causes a similar increase in ${\mathrm{HCO}}_{3}^{-}$ in solution. The decrease by 12 ${}^{\circ }$ C also increases the equilibrium magnetization and reduces thermal noise, which led to an increase in the signal-to-noise ratio of about 6 %.

After introducing the electrodes into the magnet, the signal positions shifted downfield by 1.1 ppm, and line widths became significantly broader but could be reduced to about 1 Hz by shimming except for a downfield shoulder. The signal-to-noise ratio of the spectrum was reduced significantly by 68 %. As the concentration of the carbon species remained unchanged, the decrease in the signal-to-noise ratio is a combined effect of increased noise levels and a reduced quality factor, Q , of the NMR circuit caused by the conductive components. As shown in Fig. [Fig Ch1.F7]b, the main contribution is the introduction of external RF noise due to the metallic components and cables acting as a radio antenna. Coherent external RF noise in the frequency range of ${}^{13}$ C NMR at 14.1 T (150.9 MHz) is caused by mobile radio communication [Bibr bib1.bibx11]. Introducing additional connections to the setup as well as connecting the cell directly to a powered potentiostat further decreased the signal-to-noise ratio despite using shielded coaxial cables. A highly shielded setup as described in Fig. [Fig Ch1.F3] is therefore necessary to decrease RF noise originating from external sources in order to obtain signal-to-noise ratios comparable to experiments without conductive materials. Using just single elements of the shielding setup, i.e., only the copper plate for the top opening of the magnet, the silver cloth, the common ground, or the filters, does not restore the signal-to-noise ratio to original values.

As a reference for the ${\mathrm{CO}}_{2}$ -saturated electrolyte, longitudinal relaxation times and exchange rates were determined using a standard NMR tube without the electrolysis setup. In a second step the electrodes and leads were introduced but not connected. In the final step data were collected with the full electrolysis setup shown in Fig. [Fig Ch1.F3]. All results are summarized in Table [Table Ch1.T3]. The larger errors of the ${\mathrm{CO}}_{2}$ rates are caused by a low signal-to-noise ratio of the carbon dioxide signal.

**Table 3 Ch1.T3:** Relaxation and exchange times for bicarbonate and carbon dioxide without conductive materials, with the disconnected electrolysis cell and with full electrolysis setup at 10 ${}^{\circ }$ C. The full electrolysis setup included the in operando cell, connection cables, a powered potentiostat and shielding equipment. For measurements using the electrolysis setup, no electrochemical experiments were conducted.

		Without	With	With full
		conductive	electrolysis	electrolysis
		materials	cell	setup
${\mathrm{HCO}}_{3}^{-}$	$T_{1}$ (s)	18.59±0.08	18.56±0.05	12.25±0.02
	$T_{2}$ (s)	2.04±0.00	1.40±0.00	0.97±0.00
${\mathrm{CO}}_{2}$	$T_{1}$ (s)	20.15±0.59	19.55±0.42	13.99±0.65
	$T_{2}$ (s)	4.15±0.11	2.03±0.05	2.66±0.21
	$T_{\text{exc}}$ (s)	5.23±0.18	3.31±0.25	3.79±0.37

First, the changes in relaxation and exchange behavior after insertion of the electrodes are discussed. The longitudinal relaxation times for ${\mathrm{HCO}}_{3}^{-}$ and ${\mathrm{CO}}_{2}$ remain unchanged within error boundaries compared to the electrolyte without conductive material. However, the exchange time constant of the chemical equilibrium between ${\mathrm{CO}}_{2}$ and ${\mathrm{HCO}}_{3}^{-}$ decreased from 5.23 to 3.31 s. The $T_{2}$ time constant for both ${\mathrm{HCO}}_{3}^{-}$ and ${\mathrm{CO}}_{2}$ decreased after the introduction of the electrolysis cell. The faster chemical exchange between both carbon species can only be a minor contribution to the decreased $T_{2}$ values, in particular for ${\mathrm{HCO}}_{3}^{-}$ , as it is present in significantly higher concentrations than ${\mathrm{CO}}_{2}$ . Additional contributions could be caused by local motion in the vicinity of the electrode, which may exceed the mobility due to self-diffusion by several orders of magnitude [Bibr bib1.bibx6]. In the disconnected electrolysis cell setup, such an increased mixing could originate from concentration gradients of the electrolyte near the electrode surface due to double-layer formation, possibly supported by convective flow from local heating of the electrolyte near the electrode surface. Eddy currents induced in the silver metal by the RF field of the NMR pulse excitation could cause such a local heating.

The change in the exchange time is assumed to be an indirect effect of the interaction of ${\mathrm{HCO}}_{3}^{-}$ with the polarizable silver metal electrode surface. The positively charged metal surface, as observed in the absence of an external potential (Fig. [Fig Ch1.F6]a), acts as a catalytic center for the ${\mathrm{CO}}_{2}/{\mathrm{HCO}}_{3}^{-}$ equilibrium reaction by stabilization of intermediate compounds and thus decreases the exchange time. Catalytic acceleration of the ${\mathrm{CO}}_{2}/{\mathrm{HCO}}_{3}^{-}$ equilibrium is well known for biological systems in the form of the carbonic anhydrase enzymes, which stabilize the negatively charged oxygen atoms by metal cations in a similar way during the ${\mathrm{CO}}_{2}/{\mathrm{HCO}}_{3}^{-}$ exchange reaction and increases the reaction rate by 6 to 7 orders of magnitude [Bibr bib1.bibx35]. The interaction of ions with metal surfaces by induction of dipoles is reported in the literature to extend up to 1 nm from the metal [Bibr bib1.bibx49], which can be regarded as insignificant in causing the observed changes. However, in combination with an increased mixing between surface and bulk species within the sample tube, as presumed from the $T_{2}$ alterations, such an effect could be amplified.

Secondly, changes after connection of the full electrolysis setup are examined. During the measurements employing the full in operando electrolysis setup, the cell was connected to the potentiostat. The potentiostat was powered on, but no electrochemical experiment was conducted. Therefore the cell operates in a open circuit voltage (OCV) mode with no current flow between electrodes but the voltage continuously measured by the potentiostat. Compared to the experiments with the disconnected electrolysis cell, a variation of the exchange time constant between carbon dioxide and bicarbonate cannot be precluded but is within error. However, the longitudinal ${}^{13}$ C relaxation time constant for bicarbonate and ${\mathrm{CO}}_{2}$ and the transverse relaxation time constant for bicarbonate were found to be smaller. As the experimental setup inside the sensitive volume of the NMR coil remained unchanged, the leads and filters as well as the potentiostat may be the driving forces for the increased relaxation rates. Even though the continuous voltage measurements by the powered potentiostat cause a minuscule current flow between the cell and the potentiostat, this should not have a considerable influence on the double-layer formation and the mobility of the electroactive species, since the potentiostat input is terminated with high impedance. Furthermore, it is improbable that increased stochastic fluctuations of magnetic fields originating from the potentiostat are causing such an increase in the relaxation rates. While powering the potentiostat induces increased RF noise in the NMR experiment, these fluctuations are successfully removed by the filters described in Sect. [Sec Ch1.S2.SS3].

A more probable source of the altered relaxation behavior is the changed capacity of the electrode assembly. Cables and filters can contain or act as capacitors and can provide additional mass, which changes the capability of the setup to dissipate or provide electrons at the electrodes. As OCV is an electrostatic mode of operation, the assembly may act as an additional supply or sink of electrons and thus affect double-layer formation. This in turn may affect the whole electrolyte, e.g., by changing the equilibrium between the ionic species, which may alter the pH of the system. This is known to sensitively affect relaxation properties for aqueous carbonate solutions [Bibr bib1.bibx42]. While a detailed analysis of these processes is outside the scope of the study, it highlights the sensitivity of ${}^{13}$ C NMR to investigate fundamental processes occurring during ${\mathrm{CO}}_{2}$ electrolysis, thereby justifying the efforts necessary to achieve sufficient sensitivity and resolution for in operando experiments. It also demonstrates the importance for a properly designed electrolysis setup and measurement protocol to avoid unwanted side-effects.

The results also show that the measurement setup may affect an electrochemical system. It can influence the state of the electrodes and thus their interactions with the ${\mathrm{CO}}_{2}$ -saturated electrolyte. The NMR measurements with disconnected and connected electrolysis setups show that the necessary equipment for electrochemical testing may affect the equilibrium state of the electrolysis. This is particularly pronounced at very low current densities or at OCV.

### 4.3 In operando NMR of the OCV evolution

The ${}^{13}$ C NMR spectra of the aqueous ${\mathrm{HCO}}_{3}^{-}/{\mathrm{CO}}_{2}$ sample during OCV and the potential between the working and reference electrodes are shown as a function of time in Fig. [Fig Ch1.F8]. The current density between the working and counter electrodes remains fixed at 0 mA cm ${}^{-2}$ during measurements. Therefore, no gaseous products were formed during this study. During the first 5 h of the experiment the potential drops from - 31 to - 42 mV vs. Ag / AgCl. After 12 h the potential plateaued at - 45 mV vs. Ag / AgCl and approaches equilibrium of - 47 mV vs. Ag / AgCl after 17 h.

**Figure 8 Ch1.F8:**
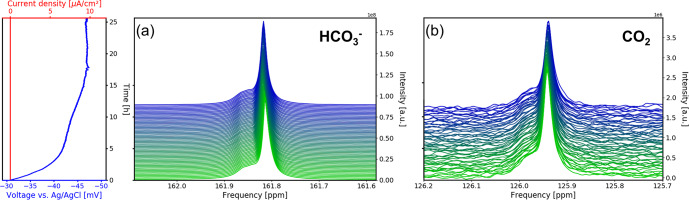
Time evolution of the ${}^{13}$ C signals for ${\mathrm{HCO}}_{3}^{-}$ **(a)** and ${\mathrm{CO}}_{2}$ **(b)** during the OCV stage vs. electrochemical potential between the working and reference electrodes and current density between the working and counter electrodes. In each subpanel the time-dependent potential and current density are shown on the left, with the corresponding spectra given on the right. After a relaxation period the potential remains at a stable at 47 mV.

The ${}^{13}$ C NMR resonances of ${\mathrm{HCO}}_{3}^{-}$ and ${\mathrm{CO}}_{2}$ remained at the initial position compared to the reference measurements. A narrow main resonance with a broader shoulder persisted throughout the OCV stage. Fitting both signals to a Lorentzian line shape, a peak separation of 0.04 ppm (6.1 Hz at 14.1 T) is obtained. The shoulder is assumed to be caused by $B_{0}$ field distortions in the proximity of the working electrode, which cannot be corrected by shimming.

**Figure 9 Ch1.F9:**
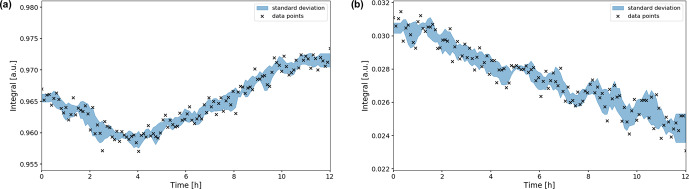
Time evolution of the ${\mathrm{HCO}}_{3}^{-}$ **(a)** and ${\mathrm{CO}}_{2}$ **(b)** signal integrals during the OCV stage. The integrals were normalized to their maximum values during the in operando experiment. Error boundaries are shown in blue. The ${\mathrm{HCO}}_{3}^{-}$ signal fluctuates within the 1 % range, while the ${\mathrm{CO}}_{2}$ signal decreases significantly in intensity over the 12 h period, down to 78 % of its maximum value.

The ${\mathrm{HCO}}_{3}^{-}$ signal shifted downfield about 0.007 ppm during the first 5 h (Fig. [Fig Ch1.F8]a), whereas the ${\mathrm{CO}}_{2}$ signal shifted by 0.002 ppm (Fig. [Fig Ch1.F8]b). Therefore, the evolution of the two signal positions appears not only to be caused by extrinsic factors such as a magnet drift. After 12 h, the ${\mathrm{HCO}}_{3}^{-}$ signal integral has increased by ca. 1 % compared to the initial intensity (Fig. [Fig Ch1.F9]a). The evolution of the ${\mathrm{HCO}}_{3}^{-}$ signal position and the intensity imply an evolution of the ${\mathrm{CO}}_{2}/{\mathrm{HCO}}_{3}^{-}/{\mathrm{CO}}_{3}^{2-}$ equilibrium since a higher chemical shift is associated with an increase in the ${\mathrm{CO}}_{3}^{2-}$ concentration [Bibr bib1.bibx1].

The intensity of the ${\mathrm{CO}}_{2}$ peak continuously decreases during the OCV stage. After 12 h the ${\mathrm{CO}}_{2}$ signal integral decreased to 78 % of the initial value (Fig. [Fig Ch1.F9]b). Using 55.0 mM as the initial concentration, as was determined in the reference experiment, this equals a concentration of 42.9 mM. After 25.6 h of OCV, the ${\mathrm{CO}}_{2}$ signal intensity decreased to 62 % (34.1 mM). This is unexpected as no ${\mathrm{CO}}_{2}$ has been converted by electrolytic processes during the OCV stage.

Leakage of ${\mathrm{CO}}_{2}$ gas during the NMR experiment is unlikely in these amounts. Permeation of carbon dioxide through the polypropylene tube cap or the glue used for sealing can be excluded, as the ${\mathrm{CO}}_{2}$ gas permeability for these materials is low [Bibr bib1.bibx22]. Furthermore, any ${\mathrm{CO}}_{2}$ loss should be compensated by the ${\mathrm{CO}}_{2}/{\mathrm{HCO}}_{3}^{-}$ equilibrium reaction, thus decreasing the ${\mathrm{HCO}}_{3}^{-}$ concentration. However, no sustained decrease in ${\mathrm{HCO}}_{3}^{-}$ concentration was observed. Furthermore, the total amount of all carbon species is unchanged after 12 h of OCV. Therefore, no ${\mathrm{CO}}_{2}$ was lost from the setup.

These observations indicate that the ${\mathrm{CO}}_{2}$ -saturated electrolyte is not at equilibrium in the initial state of the experiment. Directly after preparation, the pH value of the electrolyte was 8.2±0.15 . Given a total concentration of 1.87 M for all carbon species, the equilibrium concentration of solvated ${\mathrm{CO}}_{2}$ at that pH value is 33.7 mM. Therefore, the initial ${\mathrm{CO}}_{2}$ concentration of 55.0 mM is above the equilibrium value. The ${\mathrm{CO}}_{2}/{\mathrm{HCO}}_{3}^{-}$ system approaches equilibrium at the end of the OCV experiment, where the ${\mathrm{CO}}_{2}$ concentration equals 34.1 mM.

All changes in the ${\mathrm{HCO}}_{3}^{-}$ and ${\mathrm{CO}}_{2}$ signal integrals and the ${\mathrm{HCO}}_{3}^{-}$ signal position occur in accordance with the variations of the potential during OCV. Changes in ${\mathrm{HCO}}_{3}^{-}$ and ${\mathrm{CO}}_{2}$ signal are associated with a shift in the electrochemical potential during the OCV stage caused by an evolution of the ${\mathrm{CO}}_{2}/{\mathrm{HCO}}_{3}^{-}/{\mathrm{CO}}_{3}^{2-}$ concentrations towards equilibrium.

The relaxation and exchange time constants of ${\mathrm{CO}}_{2}$ and ${\mathrm{HCO}}_{3}^{-}$ during the OCV stage are given in Table [Table Ch1.T4].

**Table 4 Ch1.T4:** Relaxation and exchange time constants for bicarbonate and carbon dioxide during OCV. Experiments were conducted after the initial 12 h OCV period.

	$T_{1}$ (s)	$T_{2}$ (s)	$T_{\text{exc}}$ (s)
${\mathrm{HCO}}_{3}^{-}$	11.80±0.03	0.78±0.01	2.65±0.28
${\mathrm{CO}}_{2}$	13.18±0.71	2.15±0.25	

Compared to the reference measurement of the in operando cell, the exchange time decreased after the system approached equilibrium. The decrease in the exchange time is linearly proportional to the decrease in ${\mathrm{CO}}_{2}$ concentration. The decreased exchange time between ${\mathrm{CO}}_{2}$ and ${\mathrm{HCO}}_{3}^{-}$ affects the transverse relaxation process and decreases $T_{2}$ time constants, as discussed for the reference measurements. $T_{1}$ decreased only slightly as a result of the change in equilibrium and are overall comparable to the test of the in operando setup. Slight decreases in relaxation times may be the result of small variations in the electrolysis setup assembly (see reference measurements).

## 5 Conclusions

This study presented a setup for the in operando NMR study of the electrochemical ${\mathrm{CO}}_{2}$ reduction, specifically designed to observe changes in molecular dynamics in proximity to the working electrode. It was shown that ${}^{13}$ C relaxation, exchange rates, and chemical shifts can be used to sensitively characterize an electrochemical system. A key feature of the setup is the suppression of noise and external radio frequency signals introduced by conductive materials, enabling the observation of low-concentration species. Relaxation and exchange experiments provide a sensitive probe for the interaction of ionic species with metal electrodes under different electrochemical conditions. The results indicated that the electrochemical measurement equipment itself may affect the reaction and molecular dynamics. A quantitative interpretation of the data requires careful step-by-step reference measurements and a distinction between intrinsic effects caused by the investigated electrochemical system and extrinsic effects induced by the electrolysis setup. In operando NMR was employed to monitor an aqueous ${\mathrm{CO}}_{2}/{\mathrm{HCO}}_{3}^{-}$ system for electrolytic ${\mathrm{CO}}_{2}$ reduction at open circuit voltage, revealing that an (electro-)chemical equilibrium in solution evolves for a considerable time after sample preparation.

## Data Availability

All data reported in this work are available from the authors by request.

## Code availability

The simulation code used in this work is available from the authors by request.
